# Supplementary material for: Normal faulting and viscous buckling in the Tibetan Plateau induced by a weak lower crust
Source: Nat Commun. 2018 Nov 23;9:4952. doi: 10.1038/s41467-018-07312-9 (PMC6251872; doi:10.1038/s41467-018-07312-9)
Supplement: Supplementary file 1 — Supplementary Information [file 41467_2018_7312_MOESM1_ESM.pdf]

Supplementary Information for "Normal faulting and viscous buckling in the Tibetan Plateau induced by a weak lower crust" by Sarah H. Bischoff and Lucy M. Flesch.

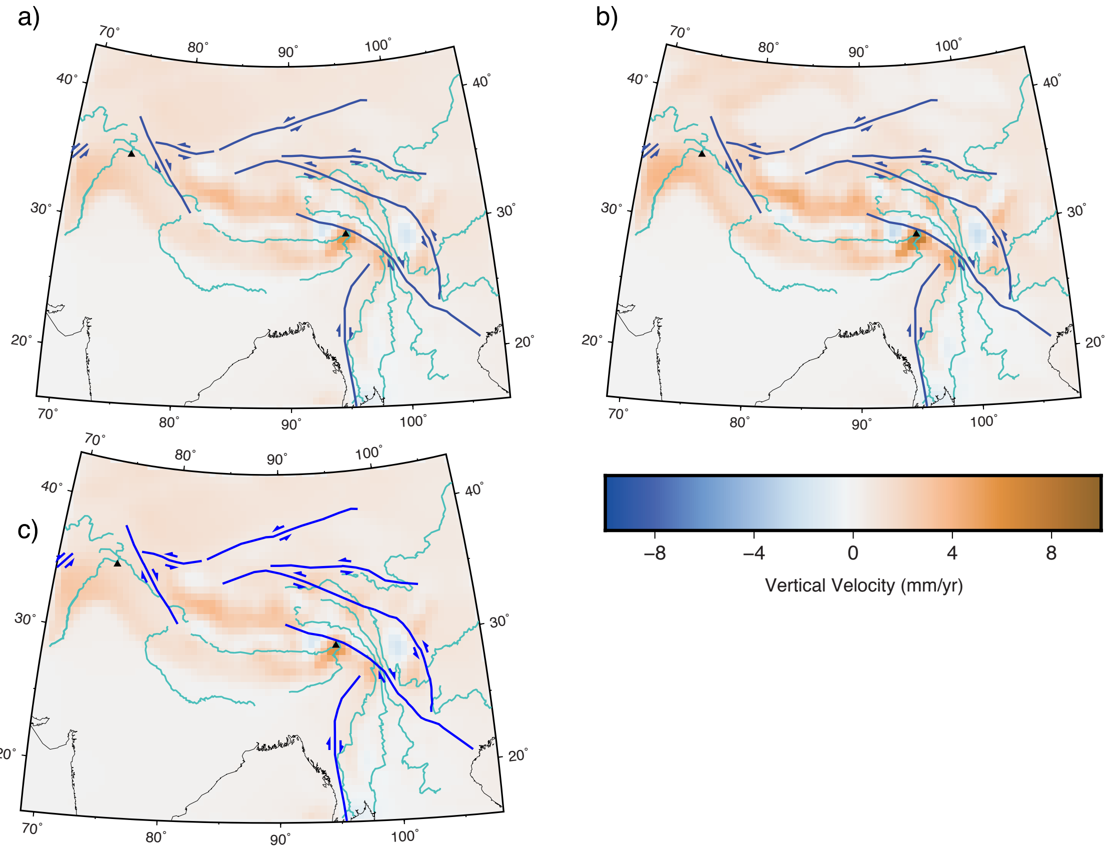

Supplementary Figure 1. Modeled solutions of vertical surface motion for block viscosity cases (a, b) and variable lower crustal thickness (c). Model solutions with color scale representing surface vertical velocity, teal lines/black symbols marking major rivers/faults, and black triangles denoting locations of Nanga Parbat and Namche Barwa peaks for cases 1 (a) and 2 (b), and for the case with a constant lower crustal layer from 20-50 km depth(c).

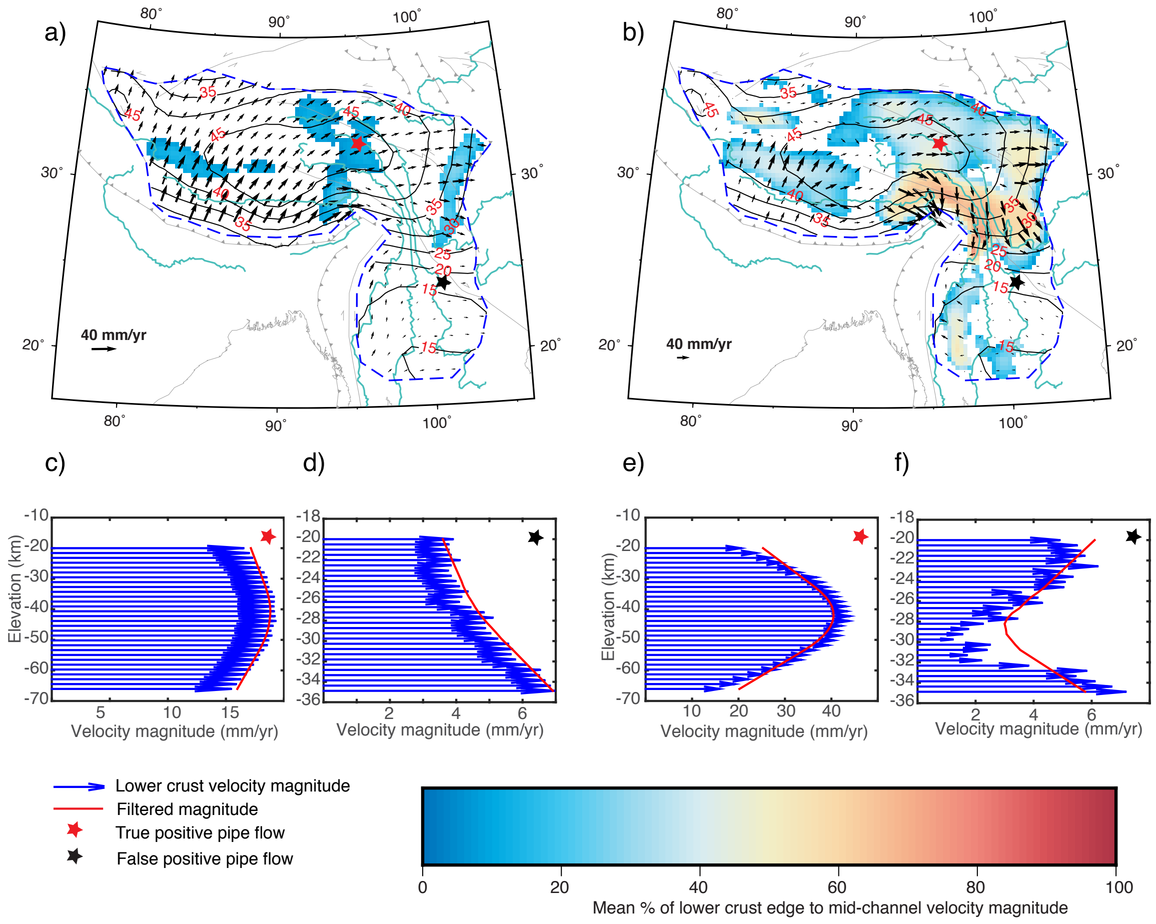

Supplementary Figure 2. Model-predicted Poiseuille flow in weak lower crust. Scale and location of pipe (Poiseuille) flow predicted by lower crustal viscosities of **a** 10^{20}, and **b** 10^{19} Pa•s. Blue-red color scale represents excess percent of mid-lower crust magnitude in relation to mean of layer top and bottom (see Methods). Dashed blue line denotes lateral bounds of weak lower crust. Black contours/red labels show thickness of weak lower crust. Black arrows show model-predicted, mid-lower crust velocities. **c-d (e-f)** Blue arrows show depth-profiles of lower crust velocity at starred locations shown in **a** (**b**). Red lines denote filtered magnitudes.

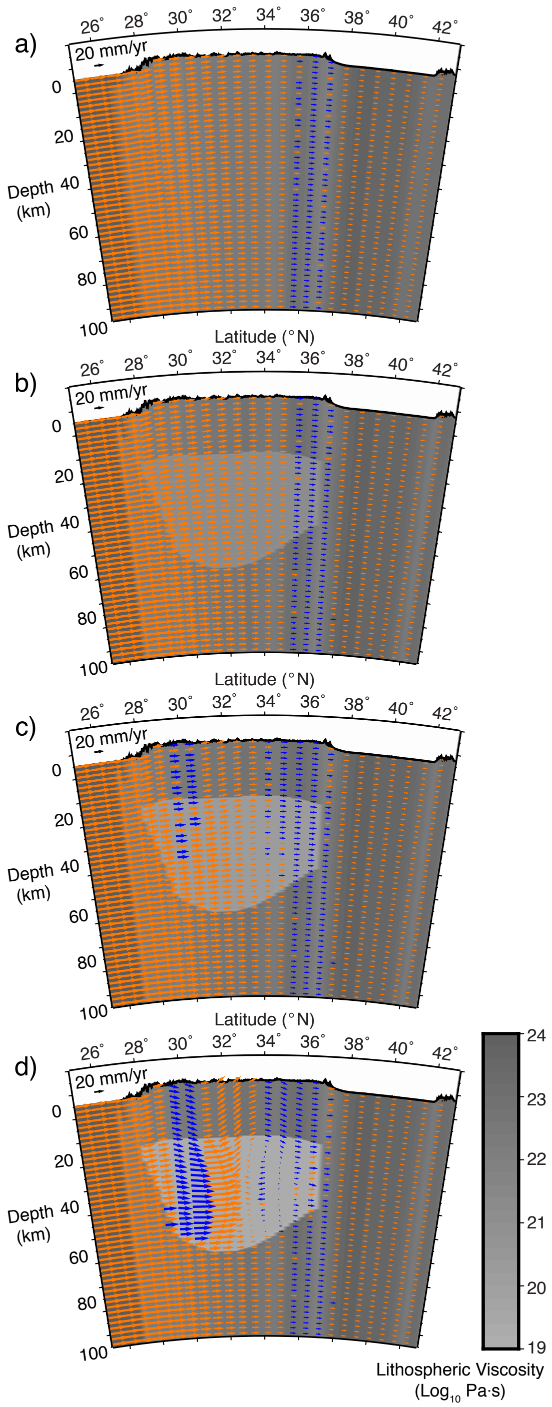

Supplementary Figure 3. Modeled velocity solutions along vertical profile at 85°E north of India. Velocity solutions for test cases with lower crust characterized by **a** no weak lower crust, **b** 10^{21}, **c** 10^{20}, and **d** 10^{19} Pa•s. Arrows are color-coded according to whether they record positive (orange) or negative (navy) uplift rates. Grey-scale represents $\log_{10}$ of viscosity, and black line shows surface elevation.
